# Study on the path of clustering construction of rural primary schools’ aesthetic education programs from the perspective of resource integration

**DOI:** 10.1371/journal.pone.0317099

**Published:** 2025-01-08

**Authors:** Zhankun Han, Yunhu Xie

**Affiliations:** 1 College of Music, Baotou Teachers’ College, Baotou, China; 2 College of Ecology and Environment, Baotou Teachers’ College, Baotou, China; University of Johannesburg, SOUTH AFRICA

## Abstract

Aesthetic education is an important part of the overall development of students. This study analyzes the number of rural schools and enrollment, number of classes, and number of full-time teachers in those schools from 2013 to 2022, and clarifies the current challenges in aesthetic education in rural elementary schools and the path of quality improvement. It then constructs a curriculum cluster model that shares resources, teachers, management, instructional design, and practical activities, etc. The results find that (1) the number of rural primary schools in 2013–2022 shows a decreasing trend, and in 2022 there was a 45.77% decrease relative to 2013. The number of rural elementary schools in 2022 shows a decreasing trend, and the relative decrease in 2022 compared with 2013 is 45.77%. From 2013 to 2022, the number of elementary schools in rural areas was more than 50% of the total number of elementary schools. Rural elementary schools in China are scattered, the distribution rate of aesthetic education resources is low, and it is difficult to popularize high-quality aesthetic education resources. (2) The number of classes in China’s rural elementary schools decreased 30.28% from 1.139 million in 2013 to 788,000 in 2022. The small size of rural schools, small number of teaching classes, and small number of students lead to less investment in resources for schools to offer aesthetic education, and the integration of collective public art courses cannot be realized. (3) Using the model of a “two-dimensional aesthetic education curriculum cluster,” we can improve the high-quality development of aesthetic education in rural schools. (4) The design path of the aesthetic education curriculum clusters focuses on the implementation and implantation of teaching objectives, teaching design and teaching process by grade, and links interest activities, nature and social observation activities, etc. Organically with aesthetic education. This study can thereby an effective path for the improvement of the quality of aesthetic education in rural elementary schools and the high-quality development of aesthetic education in rural schools.

## Introduction

The issue of aesthetic education arises from the practical activities of people and has a close relationship with the existence and development of humanity, which is the main rationale of aesthetic education. The term “aesthetic education” was first proposed by Schiller in the 1850s after the establishment of the discipline of “aesthetics” by the German scholar Baumgarten, and then by the Chinese educator Cai Yuanpei, who introduced aesthetic education into school education and strongly supported it. Aesthetic education can be regarded as the understanding of aesthetics, “aesthetic education, also known as aesthetic education,” is a type of aesthetic concept [1], aesthetic education is the basic theory of aesthetics into a kind of active, self-beneficial, the outside world to beautify the activities [2]. The definition of this concept pays more attention to “beauty;” beauty is everywhere, no matter whether in nature or society, it is inseparable from beauty. Human beings in real life, through the exploration of beauty, can understand the connotations, characteristics, and laws of beauty, to enrich the basic theories of beauty, and the research and education of the basic theories of beauty are also the popularization and popularization of the basic theories of beauty. The study and teaching of the basic theory of beauty also popularize and promote it [3]. Therefore, aesthetic education can be regarded as an education to cultivate students’ ability to recognize, love, and create beauty, and it alludes to the form of education to cultivate students’ aesthetic interest, creativity, and artistic cultivation through art, music, dance, drama, and other artistic activities [4], also known as aesthetic education an indispensable part of the education for comprehensive development [5]. Aesthetic education can promote the moral, intellectual, and physical development of students, improve their thinking, develop their moral sentiments, enrich their knowledge, develop their intellect and inspire them to love and work creatively. Aesthetic education is becoming an important choice for a culturally strong nation and is the primary task and goal of compulsory education work in the new era [6].

In recent years, the readjustment of aesthetic education has attracted considerable attention. The country has continuously issued policies related to aesthetic education, and there is a pressing need to improve students’ aesthetic feelings and humanistic literacy. Cultivating students’ all-round development has become an important task at present. Deepening the relationship between middle school education and aesthetic education is a major challenge for middle school education in the new era, and an important vehicle for reform and innovation [7]. The focus of the balanced development of aesthetic education is in the countryside, and the difficulty is also in the countryside; in particular, especially the construction of aesthetic education programs in rural primary schools has become the focus of attention. The problems faced by rural primary schools, such as remote locations, and a shortage of teachers, are not only a challenge in rural education in China, but also a worldwide problem [8]. Therefore, scholars at home and abroad have carried out multidimensional research on the challenges in quality of education in small rural schools [9, 10], the model of aesthetic education [11], and the curriculum of aesthetic education [12]. In terms of the drivers of education cluster development, in rural primary schools, Bray M proposed that increasing the utilization of educational resources, improving the quality of curriculum and teaching, promoting teachers’ cooperative learning, and enhancing the effectiveness of management are the main positive driving forces for the development of rural primary school education [13]. Giordano E [14] and Chaikoed, W [15] proposed that owing to the geographical location of rural primary schools, the teaching and learning environments of neighboring schools should be run collectively. Schools should be collectivized to share the best teaching resources and promote teachers’ growth and teachers’ progress. In terms of the concept of aesthetic education in rural primary schools, the United States focuses on the integration of art and innovation in education, Germany advocates the coexistence of perception and experience in aesthetic education, and South Korea has formulated the “rural primary school art lecturer system” in respect of aesthetic education teachers in rural primary schools. South Korea has formulated the “art system in rural primary schools” for the teachers of aesthetic education in rural primary schools. For Fan Xianzuo [16], Wu Zhihui [17] and others, rural primary school education quality presents the difficulty of the balanced development of compulsory education, therefor, we must optimize the layout, increase investment in resources, promote the innovative allocation of educational resources (cluster development), and take other measures to strengthen the school structure. Liu Shanhuai [18], Wang Yali [19, 20], and others believe that rural primary school aesthetic education has adapted to the situation of the shortage of teachers, and the local culture courses, school art courses, and subject teaching courses are closely integrated into the local culture courses, school art courses, and subject teaching courses. courses, and subject teaching courses closely together to create integration of curriculum resources, as a way to achieve the construction of curriculum diversification.

China’s rural education system is an important part of the country’s educational endeavours, it is not only related to the growth and development of young people in the vast rural areas, but is also a key link in achieving balanced social development [21]. With China’s in-depth promotion of the strategy of rural revitalization and the change of the main contradiction in society, rural education has received more attention and investment, and China has now built the largest education system in the world, with significant progress in the development of rural education being an important growth point of this achievement [22]. With regard to the development of rural education, first of all, in terms of pre-school education, the number of kindergartens in rural areas accounts for 66.21% of the total number of kindergartens in the country, showing the rapid development and popularization of rural pre-school education. At the compulsory education stage, there has been a marked improvement in the conditions of rural schools, their teaching staff and funding. At the same time, the urbanization rates for primary education and junior middle school education have reached 79.15 percent and 87.85 percent, respectively, showing a trend of rural students moving to the cities [23].

Comprehensive research has found that existing results mainly focus on the balanced development of rural primary school education [24], and the clustering development mechanism of rural primary school aesthetic education [25]. These research results are in line with the development trend of the current era; however, clustering research is slightly insufficient for the rural primary school aesthetic education curriculum. At present, there are still some shortcomings in rural primary school education in terms of education and teaching. How to solve the shortcomings in rural primary school education and create a harmonious, positive, and stable growth environment for students is a major issue that rural primary school education educators need to explore [26]. Exploring the value of aesthetic education in rural primary school education, the necessity of applying aesthetic education in rural primary school education, exploring existing problems, designing aesthetic education curriculum plans, and finally proposing corresponding improvement paths [11] have profound significance for promoting the innovative development of education in rural primary schools and the moral, intellectual, physical, artistic, and labor development of students [27]. Based on this, this study analyzes four types of data: the number of rural schools, the number of students enrolled in rural schools, the number of classes in rural schools, and the number of full-time teachers in rural schools in China from 2013 to 2022 and clarifies the dilemma of rural elementary school aesthetic education and the path toward quality improvement. Based on the theories of educational resource allocation, school-based curriculum management, and learning communities, we constructed a curriculum cluster model for sharing resources, teachers, management, teaching design, and practical activities to provide an effective path for the improvement of the quality of aesthetic education in rural elementary schools.

## Data sources and research methodology

In this study, we collected data related to the number of rural schools, number of students enrolled, number of classes, and the number of full-time teachers in rural schools in China from 2013 to 2022 from the official website of the Ministry of Education of China (http://www.moe.gov.cn/), which conducts statistical surveys of China’s annual educational resources and other statistics’ forming a complete and authoritative set of statistics on China’s education resources.

By using Origin 9.0 statistical analysis and charting software to analyze the number of rural schools, enrollment, number of classes, and number of full-time teachers in those schools in China from 2013 to 2022, we analyzed the current situation of educational resources in urban areas, townships, and villages in China, and the trend of changes and problems over the past 10 years.

This study did not involve human subjects directly.When using public data, we follow the following principles to maintain data privacy: first, we ensure that the data comes from legitimate sources and that no illegal or unauthorized data is used; second, we do not expose personally identifiable information or infringe on personal privacy during the data analysis and release process. At the same time, when using public data to write articles, we fully recognize the ethical responsibilities involved, strictly comply with relevant laws and regulations, and respect and protect personal privacy.

## Results and analyses

### The dilemma of aesthetic education in rural primary schools

**Fig 1:**
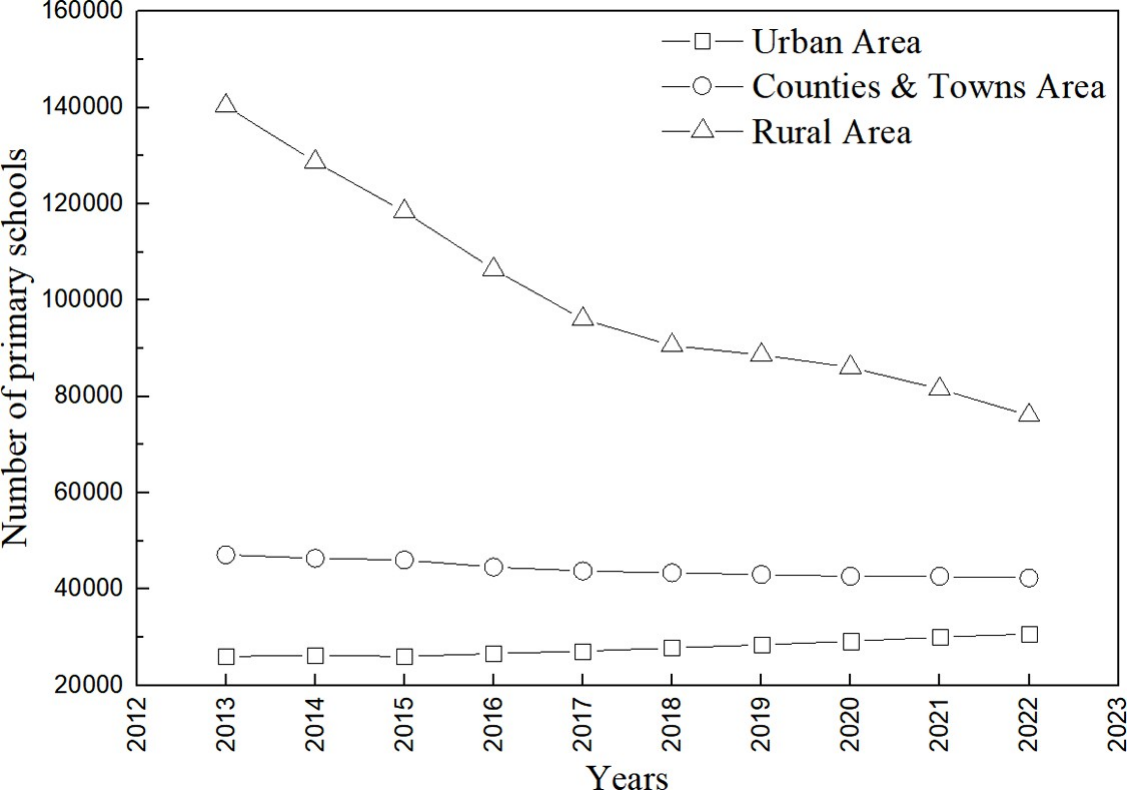
Number of primary schools, 2013–2022.

Analyzing the distribution of primary schools in urban areas, townships, and villages in China (Fig 1), the number of elementary schools in urban areas increased linearly from 2013 to 2022 (y = 546.47x − 1E + 06, R^2^ = 0.95), increasing from 26,049 in 2013 to 30,690 in 2022, a 17.82% increase. There is a linear decrease in the number of elementary schools in both townships and villages, with the decrease in the number of elementary schools in villages being the most pronounced (y = −6839.1x + 1E + 07, R^2^ = 0.93), from 140,328 in 2013 to 76,093 in 2022, a decrease of 45.77%. From 2013 to 2022, rural primary schools accounted for more than 50% of the total number of primary schools. Especially in 2013, the number of rural primary schools accounted for 65.72% of the total number of primary schools. In terms of distribution characteristics, rural primary schools are mostly located in remote scattered villages in central and western China. Rural areas in central and western China are mostly located in highlands, deserts, mountains, and hills, with dispersed settlements and inconvenient transport. As a result, many rural primary schools in China are scattered and have a low rate of distribution of aesthetic education resources, making it difficult for high-quality aesthetic education resources to be popularized and implemented in these areas.

**Fig 2:**
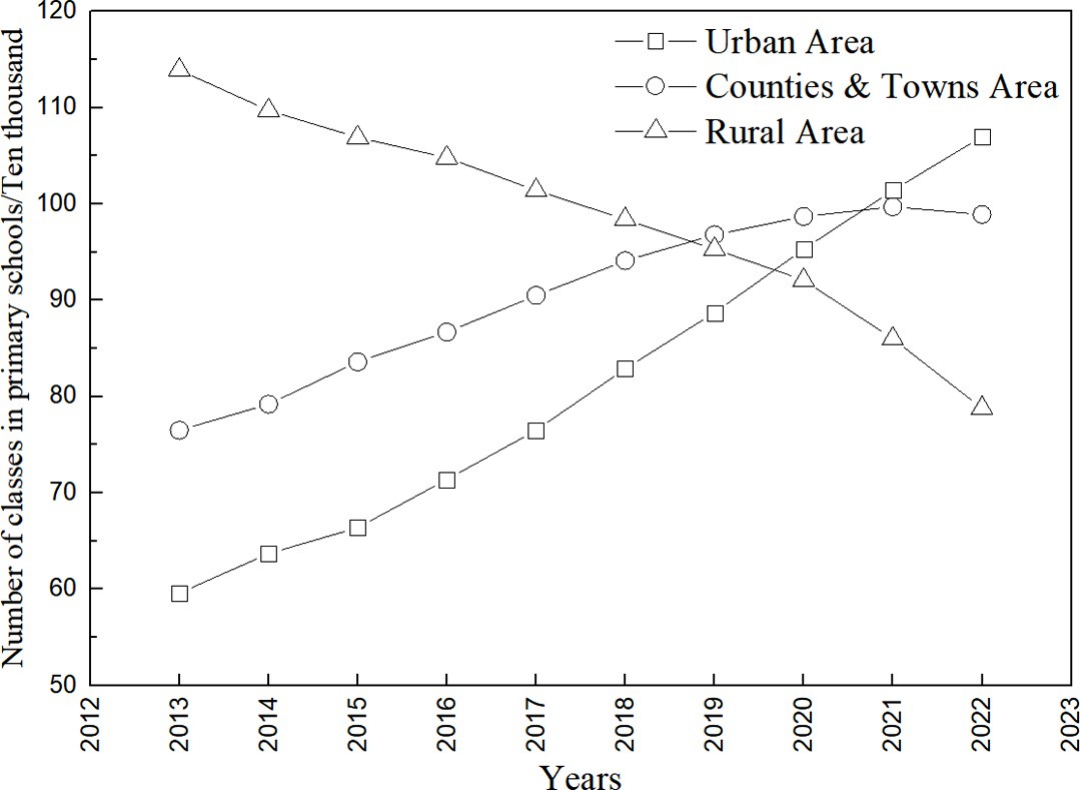
Number of primary school classes, 2013−2022.

**Fig 3:**
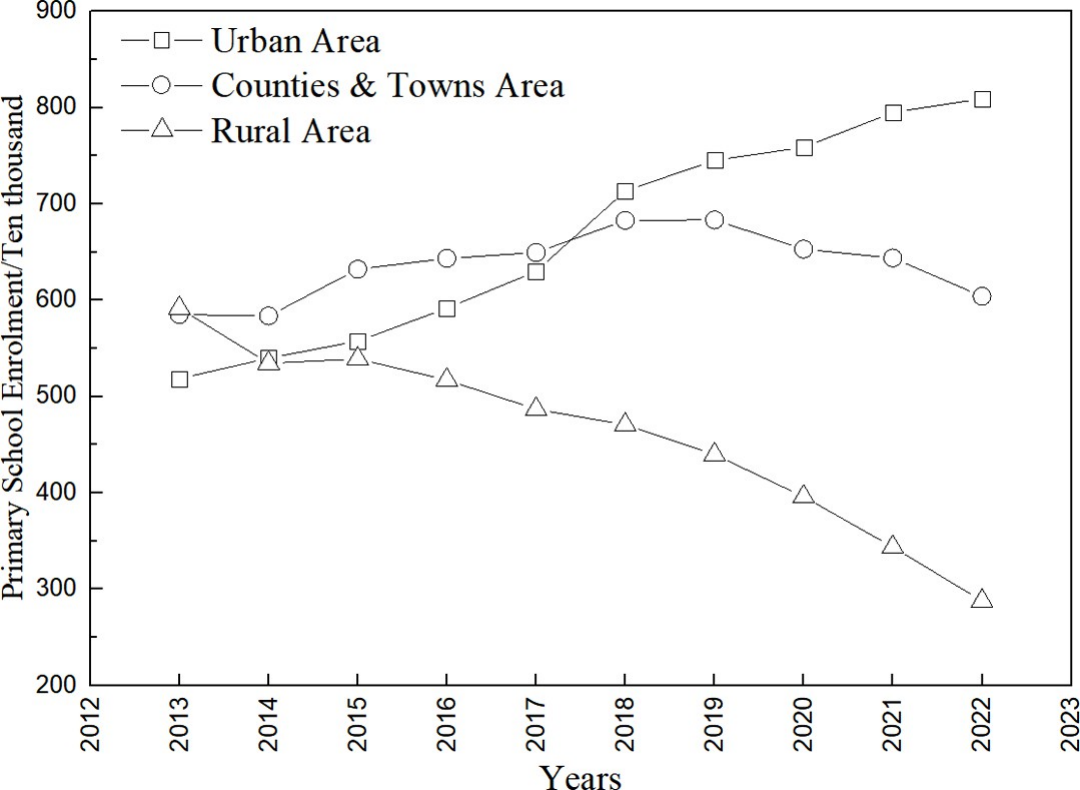
Primary school enrollment 2013−2022.

With the acceleration of China’s urbanization process, a large number of rural populations are migrating to cities. The allocation of educational resources between cities and villages is seriously uneven, and some rural primary schools have been forced to close or merge due to the conditions of operation, number of teachers, etc. The number of elementary school classes in urban areas and townships in China shows a linear increasing trend from 2013 to 2022 (linearly fitted trend lines are y = 5.4139x − 10841, R^2^ = 0.99; y = 2.7545x−5466.8, R^2^ = 0.95, respectively) (Fig 2), with a significant increase in the number of primary school classes in urban areas growing from 596,000 in 2013 to 1.07 million in 2022, an increase of 79.53%. There was a linear decrease in the number of classes in rural elementary schools (y = −3.5594x + 7279.8, R^2^ = 0.97), decreasing from 1,139,000 in 2013 to 788,000 in 2022, a decrease of 30.82% over 10 years. As can be seen from the number of primary school enrollments (Fig 3), the number of primary school enrollments in townships from 2013 to 2022 shows a trend of increasing and then decreasing. Enrollment in urban elementary schools tends to increase as a logarithmic function (y = 72788ln(x) − 553226, R^2^ = 0.97), from 5.185 million in 2013 to 8.094 million in 2022, an increase of 56.10%. Rural elementary school enrollment showed a significant linear decrease (y = −30.516x + 62026, R^2^ = 0.95), decreasing from 5,918,000 in 2013 to 2,879,000 in 2022, a decrease of 51.35%, resulting in the formation of many small schools with fewer than 100 students. The small size of rural schools, small number of teaching classes, and small number of students have led to low investment in resources for the provision of aesthetic education in schools, and the integration of collective public art programs has not been possible.

**Fig 4:**
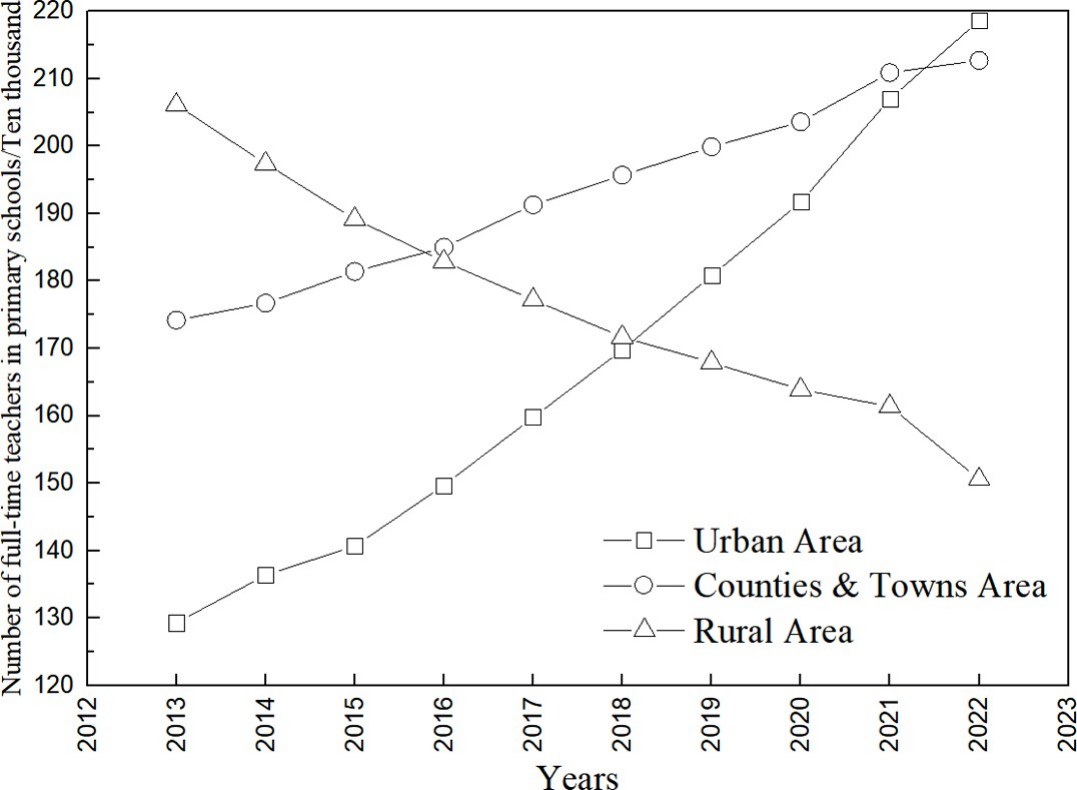
Number of full-time teachers in primary schools, 2013–2022.

**Fig 5:**
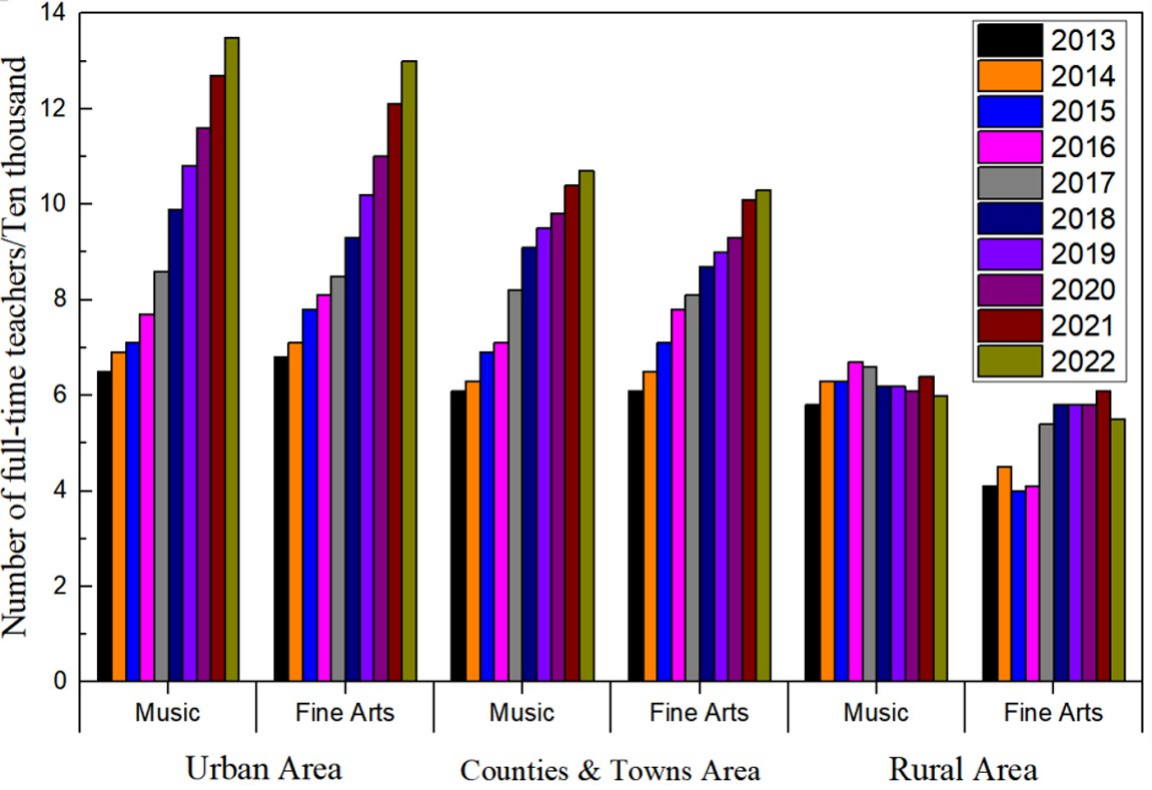
Number of full-time teachers specializing in aesthetic education in primary schools, 2013–2022.

According to the statistics on the number of full-time teachers in primary schools from 2013 to 2022 (Fig 4), there was a linear increase in the number of full-time teachers in elementary schools in urban areas and townships (linearly fitted trend lines are y = 10.038x − 20082, R^2^ = 0.98 and y = 4.5212x − 8928.4, R^2^ = 0.99, respectively), increasing from 1,293,000 and 1,742,000 in 2013 to 2,186,000 and 2,127,000 in 2022, an increase of 69.06% and 22.10%, respectively. There was a significant linear decrease in the number of full-time teachers in villages (y = −5.6273x + 11530, R^2^ = 0.98), from 206.1 in 2013 to 150.6 in 2022, a decrease of 26.93%. The curricula of rural primary schools mainly focus on language, mathematics, and physical education, and the proportion of art, music, and local culture courses offered is notably insufficient (Fig 5). Among them, the proportions of language, mathematics, and physical education courses offered are 71.3%, 69.1%, and 80.2%, while the proportions of art, music, and local culture courses offered are only 31.2%, 35.5%, and 18.7%, respectively. With a reduction in the opening rate of aesthetic education courses, many teachers of aesthetic education flow to urban schools or transfer to other industries, which also makes the post of aesthetic education teacher unattractive, and the motivation of teachers to teach is insufficient. At the same time, this has led to a decline in students’ interest in learning and a low level of satisfaction. Against this backdrop, the satisfaction rate of rural parents and students with their interest in learning at school is only 23.1%, far lower than the 63.2% satisfaction rate for interest in learning in urban schools.

### The practical path of clustering the aesthetic education curriculum in rural primary schools

**Fig 6:**
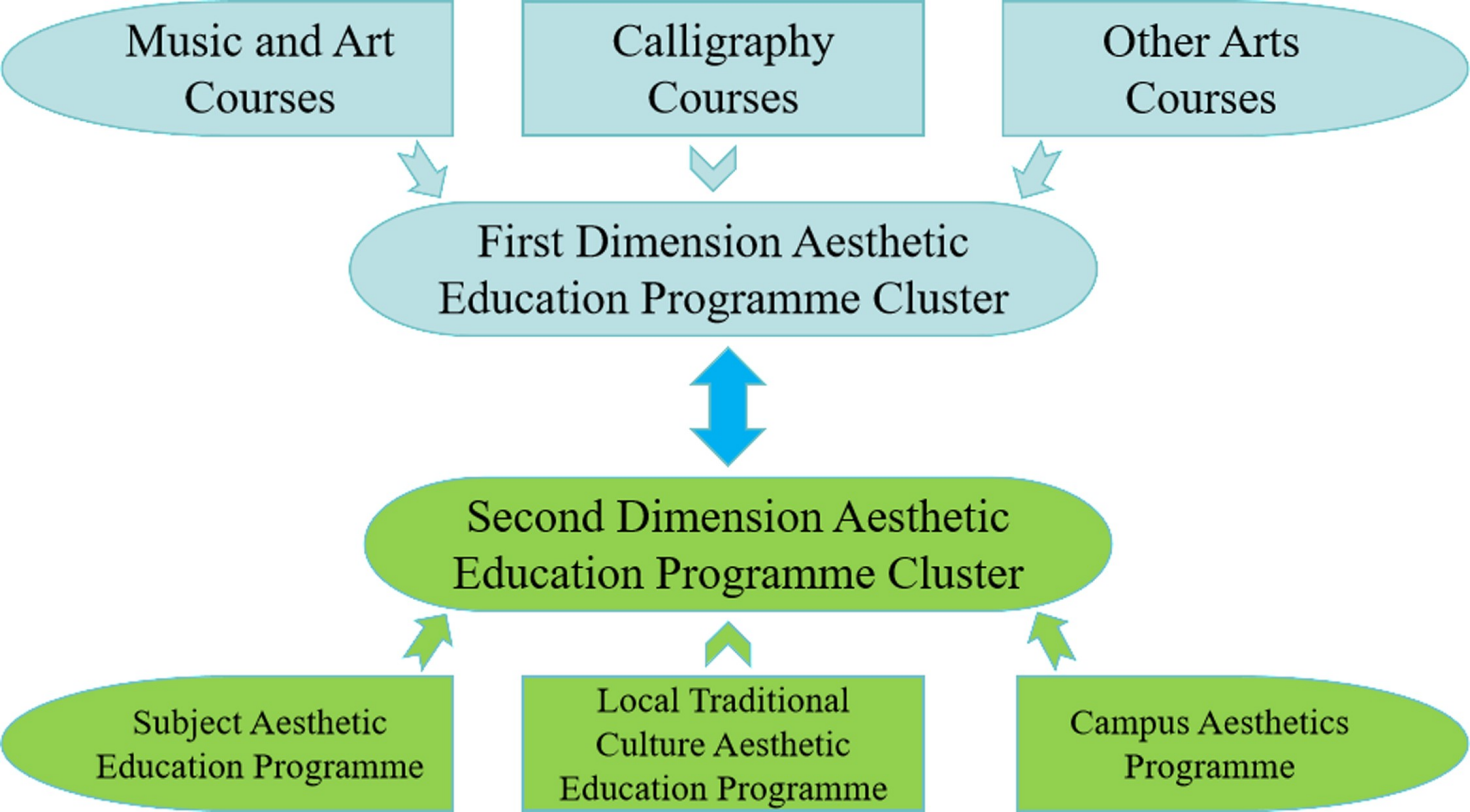
Cluster structure of 2D aesthetic education program in rural primary schools.

At present, music and art programs in most rural primary schools are directly equivalent to aesthetic education programs, and music and art programs have been transformed into simple singing and painting technique training classes, neglecting the nurturing function of aesthetic education. Technical training in music and art has replaced aesthetic education, and aesthetic education has become a lacuna of basic education. Drawing on years of exploration and practice, this study proposes the model of a “two-dimensional aesthetic education curriculum cluster” (Fig 6) to enhance the development of high-quality aesthetic education in rural schools.

#### Level 1 aesthetic education program cluster

**Fig 7:**
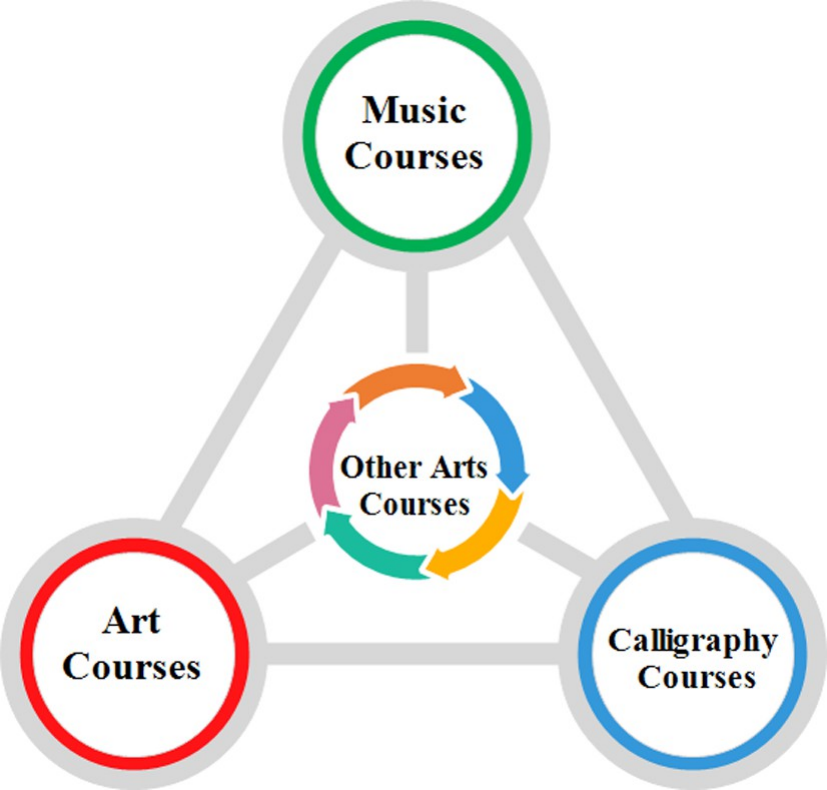
Structure of the “3+x” model for the first level of aesthetic education program clusters.

The first level of the aesthetic education curriculum cluster in rural schools is to build a basic aesthetic education curriculum based on the “3+x” model (Fig 7). The “3” refers to music, art, and calligraphy courses, while the “x” represents other art courses. The compulsory education stage enriches the content of the art curriculum, and based on a good music, art, and calligraphy course, art courses such as dance, theater, film, and television are gradually introduced. Some rural schools that are temporarily unable to offer more public art education courses because of their conditions can start by offering a good music course, art course, and calligraphy course—the three art courses and their aestheticized teaching reforms.

At present, many rural schools already offer other arts programs, for example, in the Inner Mongolia Autonomous Region, relying on the rich resources of folk songs, traditional folk songs are incorporated into the teaching of the public arts education curriculum, and in Shanxi, opera music is integrated into the school’s public arts education curriculum. At the same time, some schools provide higher-level arts training opportunities for students with special interests and strengths in the arts.

#### Level 2 aesthetic education program cluster

**Fig 8:**
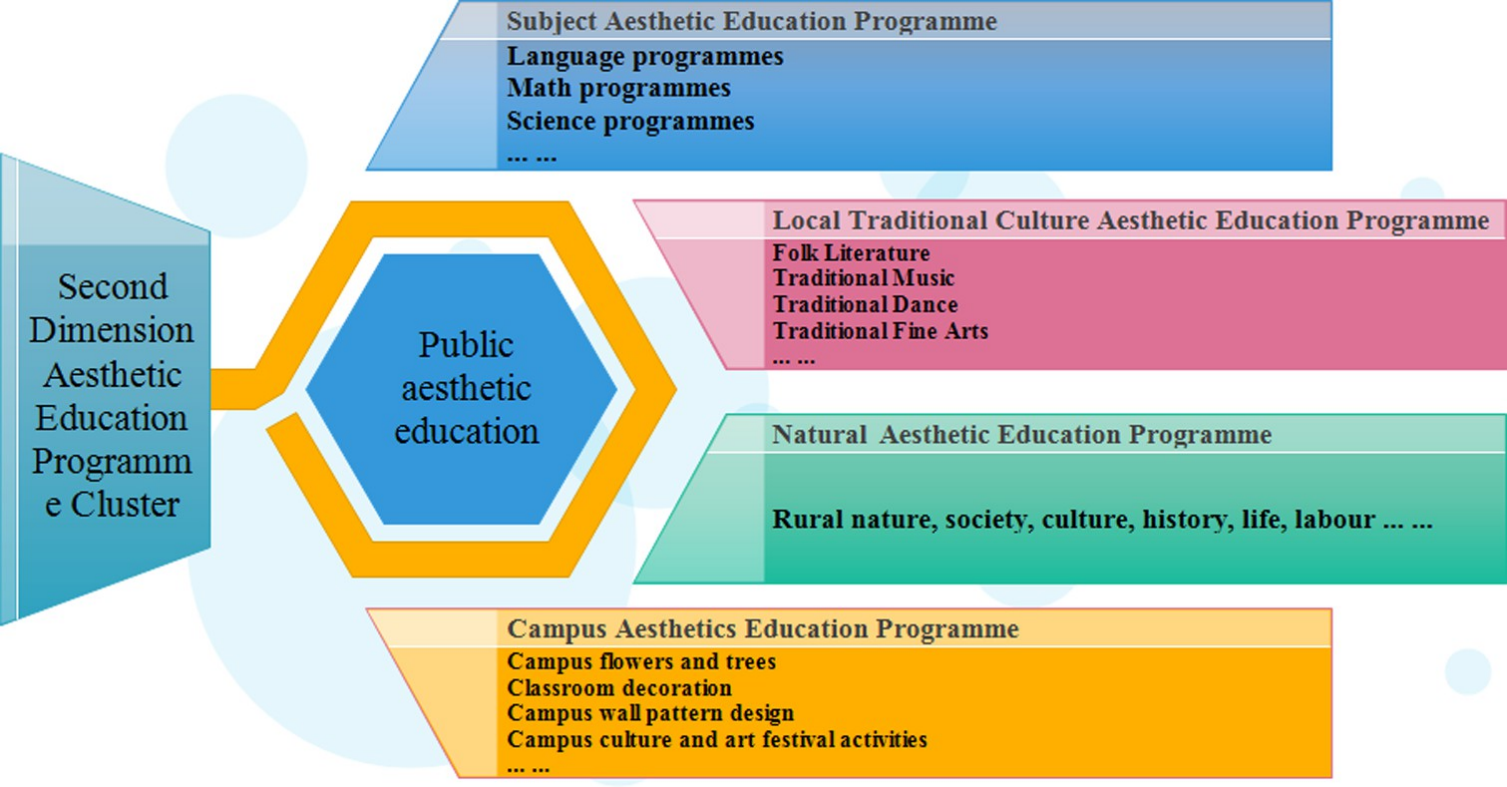
Structure of the aesthetic education programme cluster model at level 2.

The second cluster of aesthetic education programs in rural schools is based on subject aesthetic education programs, natural aesthetic education programs, local traditional culture programs, and school environment aesthetic education (Fig 8), which complement to and complete the public aesthetic education, reflecting the whole process of the aesthetic education implementation path.

(1) Subject Aesthetic Education Curriculum

Language, mathematics, and science courses originally contained aesthetic elements, including content and form. For example, the literary aesthetics of language are more humanistic, and the symmetry of mathematics is interesting, etc. Therefore, refining the aesthetic content of subjects based on daily teaching, creating aesthetic methods of subjects, and cultivating the aesthetic qualities of subjects embody the unique objectives and functions of these aesthetic courses.

(2) Local traditional culture programs

Intangible cultural heritage items distributed throughout the country have a natural function in rural aesthetic education. In the Inner Mongolia Autonomous Region, for example, there are six categories of non-heritage programs suitable for children and young people in rural schools: folk literature, traditional music, traditional dance, traditional art, traditional skills, and folklore. Rural primary schools make full use of them, actively engaging with local non-heritage resources, selecting the best and optimizing them appropriately, and integrating them into the existing aesthetic education curriculum system.

China has a huge reserve of intangible cultural heritage. To date, there are more than 100,000 items representative of intangible cultural heritage at all levels in China, and it ranks first in terms of the number of items inscribed on the UNESCO list of intangible cultural heritage of humankind. Most of these intangible cultural resources are found distributed in rural areas, and diverse intangible cultural heritage items exist in different regions, not only embodying excellent local cultures but also having natural aesthetic education functions. At the same time, we can cooperate with local intangible cultural heritage museums, art galleries, and cultural and art enterprises, relying on the advantageous resources of all parties, to offer practical experience classes and study courses on intangible cultural heritage. This will enrich the system of aesthetic education programs, to create opportunities for students to come into contact with cultural and art enterprises and intangible cultural heritage bearers, and guide students to participate in the local intangible cultural heritage exchange activities, thereby building a close connection between the aesthetic education programs and the development of regional intangible cultural heritage undertakings.

(3) Natural Aesthetic Education Curriculum

Rural small-scale schools are embedded in the rural society, so they can design aesthetic education curricula for the natural environment and agricultural labor. The natural aesthetic education curriculum includes rural nature, society, culture, history, life, and labor. These curriculum resources come from the daily life of pupils in small-scale rural schools, have a natural connection with children’s experiences, and are familiar to them. Teachers can introduce these resources into their teaching practice, establish an organic connection between the scientific world and the empirical world, and help students to gain meaningful learning so that natural aesthetic education is carried out throughout the daily life of rural children.

(4) Aesthetic Education in Campus Culture

The optimal design of campus culture affects students’ aesthetic habits directly. The aesthetic education teaching content of campus culture includes campus flowers and trees, classroom layout, campus wall pattern design, and campus culture and art festival activities. Among these, campus culture and art festivals have typical aesthetic education importance in primary schools. For example: every semester over four seasons, the schools held a science and technology festival, a sports festival, an art festival, social practice, scientific exploration, and other activities, In the process of organizing and implementing campus art, teachers take over the use of language, art, information technology, and other disciplines of knowledge and skills, and guide the students in the activities of the design of illustrated posters, invitations, cards, etc., so that in the cross-disciplinary learning the students experience beauty, create beauty, and improve their aesthetic literacy and creativity. When teachers tap into these educational resources, they find an entry point for the integration of aesthetic education and interdisciplinary teaching.

However, the “Two-dimensional Aesthetic Education Curriculum Cluster” model proposed in this study will inevitably face certain challenges in the process of being tested, the first of which is whether or not the resources of the aesthetic education curriculum in the rural schools in various regions are well allocated, especially in some remote areas, where the implementation of the curriculum may be limited due to the lack of necessary teaching facilities and materials; secondly, the lack of teachers is also an important limiting factor for the implementation of the aesthetic education curriculum in rural schools. Secondly, the lack of teachers is also an important limiting factor for the implementation of the aesthetic education program in the rural schools, as the majority of the aesthetic education teachers choose towns and cities as the first place of choice, and very few of them are willing to go to the villages to teach in the first place, which results in the imbalance of teachers between towns and villages, and limits the implementation of the beauty and aesthetics curriculum.

### Pathways for implementing aesthetic education program clusters in rural schools

The quality education system for students in rural schools in the new era lies in meeting the demand for the era of “aesthetic education +,” and aesthetic education in rural schools has new characteristics and challenges, so we will integrate the concept of “secondary aesthetic education program cluster” into the teaching objectives of aesthetic education and study the educational significance of aesthetic education in the growth process of children of school age in rural areas. Thus, we can respond better to the discourse of the teaching reform of the new era, which is to incorporate the “five education” into the teaching materials and curricula of children and youth. Therefore, it is of great practical significance that the cluster of practical courses should be used to guide the design, implementation and reform of teaching, with an emphasis on stimulating the potential and ability of each student.

**Fig 9:**
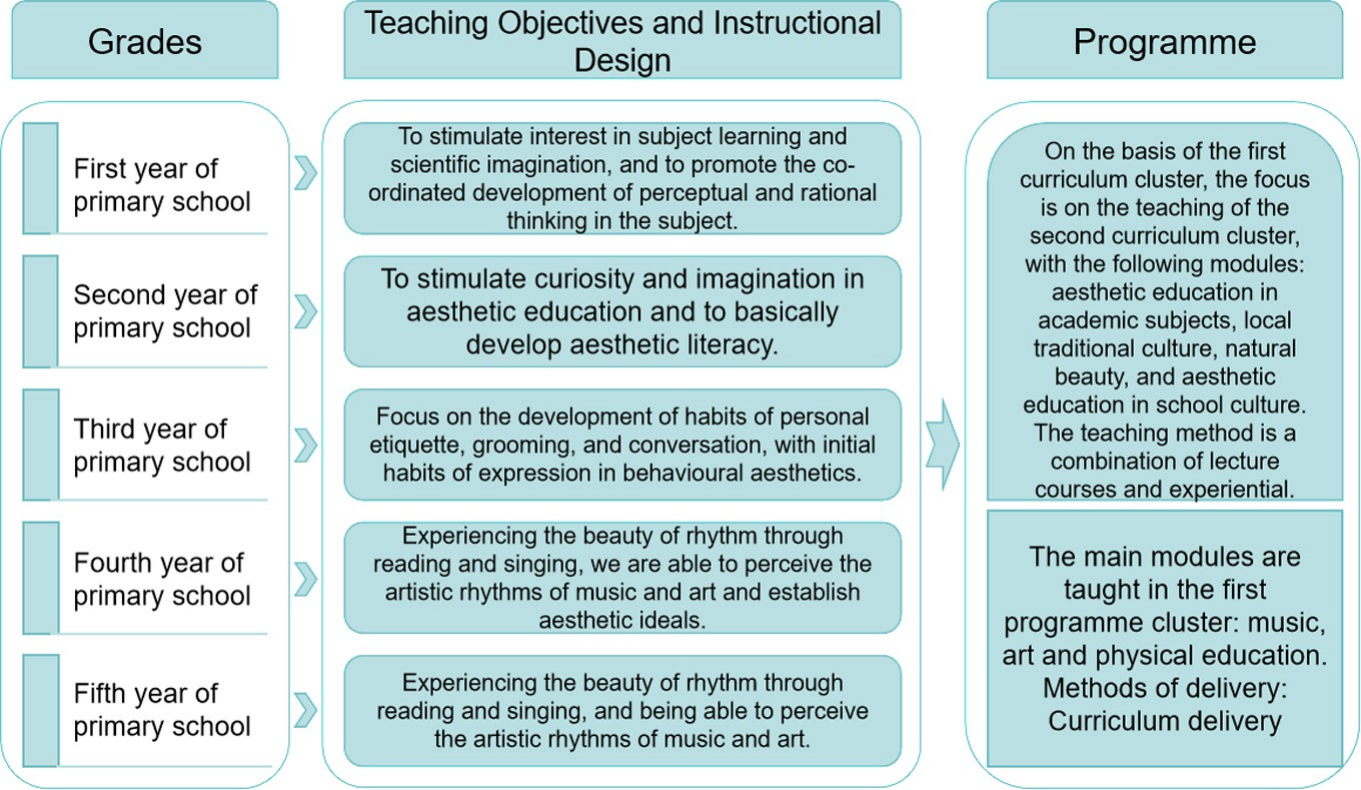
Flowchart for teaching and learning implementation of the 2D aesthetic program cluster in rural schools.

The age group of children and teenagers in rural schools focuses on the age group of 7–12 years old, where students are in the age of development, and the cognitive level of children in each age group is different. Therefore, the design path of integrating the aesthetic education curriculum clusters into teaching focuses on the implementation of the teaching objectives, the design of the teaching, and the teaching process by grade level (Fig 9). The interest activities of nature and social observation, arts and sports, science and technology, games, and the construction of school environment beautification are linked organically with aesthetic education.

## Conclusions

1. Rural primary school aesthetic education is faced with the problems of a large number of schools, dispersed locations, and a low distribution rate of aesthetic education resources. There are many small-scale schools with fewer than 100 students in rural areas, and it is difficult to guarantee the quality of the student population. The low rate of distribution of resources for aesthetic education makes it difficult to popularize quality aesthetic education resources.
2. The number of elementary school classes in China’s villages from 2013 to 2022 decreased from 1,139,000 in 2013 to 788,000 in 2022, a decrease of 30.82%. Small size of rural schools, small number of teaching classes, and small number of students lead to less investment in resources for schools to offer aesthetic education, and the integration of collective public art courses cannot be realized.
3. Using the model of “two-dimensional aesthetic education curriculum clusters,” the development of high-quality aesthetic education in rural schools can be improved. The design path for the integration of aesthetic education curriculum clusters into teaching focuses on grade-level implementation and implantation of teaching objectives, teaching design and teaching processes.

## Policy recommendations and future prospects

Resource constraints and insufficient teachers are two major problems in promoting the “two-dimensional aesthetic education curriculum clusters”. In order to solve these problems, the following suggestions are made:

1. It is recommended that local governments increase their investment in rural aesthetic education, especially in the cultivation and introduction of aesthetic education teachers. At the same time, digital technology is utilized to broaden the channels of access to educational resources, and quality aesthetic education courses are shared through online platforms to make up for the limitations of physical space. In addition, establishing an exchange mechanism between urban and rural schools to promote resource sharing and experience sharing is also an effective way to improve the quality of rural aesthetic education.
2. We can explore the establishment of a hand-in-hand support mechanism between colleges and universities and primary and secondary schools, and encourage famous teachers of aesthetic education to enter the countryside and rural students to enter urban art venues to promote resource sharing and exchange. At the same time, utilizing digital technology to empower aesthetic education is also an effective way. For example, relying on the National Intelligent Education Public Service Platform to develop high-quality aesthetic education digital education resources, and utilizing cloud exhibitions, virtual performances and other technological means to enrich teaching content and improve teaching quality.
3. The “two-dimensional aesthetic education curriculum clusters” model has injected new vitality into aesthetic education in rural schools, giving every child the opportunity to come into contact with and feel the power of beauty through innovative curriculum design and practical activities. This not only improves the quality of education in rural schools and the aesthetic quality of children, but also lays a solid foundation for rural revitalization and cultural confidence. In the future, we look forward to more innovations and explorations that can help every child fly freely in the world of beauty.

## Supporting information

S1 File (XLS)
